# Filial cannibalism of *Nabis pseudoferus* is not evolutionarily optimal foraging strategy

**DOI:** 10.1038/s41598-024-59574-7

**Published:** 2024-04-19

**Authors:** József Garay, Manuel Gámez, Yohan Solano-Rojas, Inmaculada López, Ana Belén Castaño-Fernández, Zoltán Varga, Tamás F. Móri, Villő Csiszár, Tomás Cabello

**Affiliations:** 1 HUN-REN Centre for Ecological Research, Institute of Evolution, Konkoly-Thege M. út 29–33, 1121 Budapest, Hungary; 2 Research Centre for Mediterranean Intensive Agrosystems and Agrifood Biotechnology (CIAMBITAL), Agrifood Campus of International Excellence (CEIA3), University of Almeria, Ctra. de Sacramento S/N, La Cañada de San Urbano, 04120 Almería, Spain; 3 Department of Mathematics, University of Almería, Ctra. de Sacramento S/N, La Cañada de San Urbano, 04120 Almería, Spain; 4 Department of Mathematics and Modelling, Institute of Mathematics and Basic Science, Hungarian University of Agriculture and Life Sciences, Páter K. u. 1, Gödöllő, 2100 Hungary; 5 HUN-REN Alfréd Rényi Institute of Mathematics, Reáltanoda u. 13-15, Budapest, 1085 Hungary; 6 Department of Probability Theory and Statistics, Eötvös Loránd University, Pázmány Péter s. 1/C, Budapest, 1117 Hungary

**Keywords:** Long-term growth rate, Reproductive season growth rate, Life reproductive success, Numerical response, Filial cannibalism, Optimal foraging, Time constraint, Ecological modelling, Entomology

## Abstract

Using a recursion model with real parameters of *Nabis pseudoferus,* we show that its filial cannibalism is an optimal foraging strategy for life reproductive success, but it is not an evolutionarily optimal foraging strategy, since it cannot maximize the descendant’s number at the end of the reproductive season. Cannibalism is evolutionarily rational, when the number of newborn offspring produced from the cannibalized offspring can compensate the following two effects: (a) The cannibalistic lineage wastes time, since the individuals hatched from eggs produced by cannibalism start to reproduce later. (b) Cannibalism eliminates not only one offspring, but also all potential descendants from the cannibalized offspring during the rest of reproductive season. In our laboratory trials, from conspecific prey *Nabis pseudoferus* did not produce newborn nymphs enough to compensate the above two effects.

## Introduction

The cannibalism is ubiquitous in animals from protists and rotifers, through invertebrates, to vertebrates^1–3^. For many arthropods cannibalism is a normal phenomenon^4,5^. First of all, cannibalism is a feeding behavior. It increases the net energy gain^6–8^, and strong resource limitation can facilitate cannibalism^2,7,9,10^. However, cannibalism is a competitive behavior, when cannibal phenotype can mainly kill non-relative conspecific, so removing potential competitors^11,12^. In this paper we are interested in whether filial cannibalism of the territorial *Nabis pseudoferus* Remane (Hemiptera: Nabidae) is an evolutionarily optimal feeding strategy or not. Since the territorial behavior precludes the competitive advantage of cannibalism, *N. pseudoferus* is one of the excellent animals to test whether its cannibalism is an optimal feeding strategy or not.

The cannibal and pure carnivore damsel bug *N. pseudoferus* is not a random searcher, since it uses search image^13–16^. It is a totally opportunistic predator^8,17,18^, which always attacks the encountered prey, independently of its actual prey preference. Since conspecifics are a high-quality resource (in the correct stoichiometric ratios^19^) for cannibals^20^, cannibalism is a beneficial feeding strategy for the female. *N. pseudoferus*. Furthermore, *N. pseudoferus* is a strictly territorial predator^8,10^. A consequence is the following trade-off on the total offspring number: the cannibal *N. pseudoferus* female can only consume its own offspring, thus it decreases the number of nymphs, while increases its own egg number.

In classical optimal foraging theory^21^, the forager maximizes its net biomass intake rate, because the numerical response is defined as the consumed biomass multiplied by the conversion rate^21–23^. Observe that the direct application of optimal foraging theory to territorial cannibal consumers is questionable, since they kill their own offspring, thus the long-term growth rate of the phenotype is necessarily not proportional to the consumed biomass. Thus here we have to consider the optimal foraging theory from the view point of evolutionary theory. Intuitively, the highly cannibal female may decrease its fitness (growth rate of its lineage). For instance, if each female has consumed the overwhelming majority of its eggs or its new hatched instars, then it decreases its fitness. However, with very small cannibalism rate, the female may increase its fitness, for instance, if each female can produce a large number of eggs from only one of its own consumed offspring. From these two extreme situations, it seems that there may exist a fitness optimizing cannibalism rate.

In evolution theory, the fitness is optimized. However, the definition of fitness is a crucial point. Here we consider two fitness definitions: the long-term growth rate of a phenotype and the life reproductive success of an individual. Recently, having used biomathematical models for overlapping generations^24,25^, we have to point out that the long-term growth rate of a phenotype is the best theoretical definition of fitness in the following sense. Let us consider a selection model of two phenotypes with overlapping generations: selfish phenotype (maximizing the life reproductive success, i.e. the average offspring number during an individual life span), and Darwinian phenotype (maximizing the phenotypic long-term growth rate). Then the latter phenotype outperforms selfish phenotype. The reason for that is the following. In the case of overlapping generations, not only the focal female produces offspring, but its offspring also has offspring (grandchildren of the focal female), thus the life reproductive success and the total number of descendants of the focal female during one reproductive season in general can take their maximum at different cannibalism rates^24,25^. We emphasize that the long-term growth rate depends on the life history of *N. pseudoferus,* namely the developmental times of nymphs and the life span of the adult. In this paper, we are interested in whether for *N. pseudoferus* the above two fitness notions give different predictions, or not.

Filial cannibalism is clearly a feeding strategy, thus at first we have to check the following hypothesis: Filial cannibalism of a *N. pseudoferus* female, from the perspective of evolution theory, can be considered as an optimal feeding strategy. Our concrete study is based on our second hypothesis: the optimal cannibalism rate of *N. pseudoferus* is fixed by natural selection. Under these hypotheses, we can calculate the cannibalism rate that maximizes the long-term growth rate of a phenotype and the life reproductive success of a female. Thus, firstly we introduce a mathematical model as close as possible to the population dynamics of *N. pseudoferus*. This dynamics takes account of different time constraints: the optimal foraging times (e.g. handling time) and the life history times (developmental time durations). Secondly, we estimate all parameters of our model by laboratory trials. Thirdly, we calculate the fitness-maximizing prey preference (i.e. optimal foraging strategy) based on the estimated parameters of *N. pseudoferus*. Finally, by comparing the prediction of the model with the experimental knowledge, we can check whether our first hypothesis is true or falls in the case of *N. pseudoferus*.

## Material and methods

We build up a mathematical recursion model, which is as close as possible firstly, to the standard deterministic modeling methodology of theoretical biology, and secondly, to the experimental methodology (please see SI A).

### Experimental data

The biological parameters used in the model were obtained from previously published works as searching time and handling time^8^ and rate of success of attacks^10^. We also estimated the still missing parameters necessary for the mathematical investigations. For this purpose, one trial was carried out under laboratory conditions (25 ± 2 °C, 65 ± 10% RH and 16:8 h of light: dark). We used fertilized adult females of *Nabis pseudoferus*. The adult females were fed, according to treatment, on heterospecific prey (larvae of II- and III-instar) *Spodoptera exigua* (Lepidoptera: Noctuidae) or conspecific prey (nymphs of II- and III-instar) of *N. pseudoferus*. The number of replications were 22 for conspecific prey and 23 for heterospecific prey. The oviposited eggs were evolved until reaching the adult stage, using, in each treatment, the same prey species that were offered to the adult females. The data collected were longevity, fecundity, and fertility of females, development times of the immature’s stages.

This made it possible to establish the life cycle of the predator species for each prey species. The data were analyzed statistically by means of generalized linear models (GZLM) using IBM SPSS statistical software version 28. (See SI, Section a.1.1.)

The parameters for *S. exigua* and *N. pseudoferus* obtained from the trial were as follows (see SI, Section a.1.1.2): *Y* = 16.8, *b* = 0.44, *a*_*3*_ = 0.91, *a*_*4*_ = 3.00, and *a*_*5*_ = 6.14. In turn, the following parameters were obtained from previously published works: *τ*_*a*_ = 23.3, *τ*_*b*_ = 16.6, and *τ*_*s*_ = 5.3^8^; *k*_*3*_ = 1.00, *k*_*4*_ = 0.80, and *k*_*5*_ = 0.55^10^.

### Theoretical model

The formal description of the mathematical model given in the SI B, is based on the following reasoning. The hypothesis we study is that from evolutionary viewpoint, *N. pseudoferus* has an optimal rate of cannibalism. In order to determine the rate of cannibalism with mathematical methods, we kept the two basic principles of classical optimal foraging theory, namely: we built up the numerical response based on the Holling type II functional response, and we used physical time for our dynamics^8^. In our model building we took into account that all model parameters should be measurable under laboratory conditions, and we really measured them. Since under natural conditions *N. pseudoferus* has overlapping generations, and the success of maternal cannibal attacks depends on the developmental stages of nymphs, we introduced a discrete time dynamics (recursion). Our recursion is an optimal foraging model built upon the evolutionary paradigm, since it makes fitness maximization possible, strictly based on the fact that in laboratory we could measure the conversion coefficients necessary for the numerical response. From entomological viewpoint, it is a necessary simplification that temperature dependence, in general, is not built into the standard theoretical models. Of course, we are aware of the fact that insects do not have constant temperature, so their individual development does not depend on the physical time, but on the temperature accumulated in a given time period^26,27^. A further complication is that other model parameters (activity time and lifetime) are also temperature dependent. Furthermore, in the habitat of *N. pseudoferus*, the yearly time variation of the temperature in subsequent years can be quite varied.

We will consider two questions. Firstly, we are interested in the *life reproductive success* of a focal female. Secondly, we ask how many descendants a focal female called Eve will have during one reproductive season (8 months from March to October, about 250 days), in other words, we are interested in Eve’s “*reproductive season growth rate*”. To do this, we have to take into account two main components: the life history of *N. pseudoferus* and its prey preference-dependent numerical response.

*N. pseudoferus* has overlapping generations. During its development, *N. pseudoferus* has 5 nymph stages with different time durations (see Table S2; section a.1.1.2 of the SI). Since our recursion is a discrete time model, we consider 5 days as a time unite and 6 different developmental cohorts: cohort 1 of eggs laid in time period $t$, cohort 2 of eggs (since hatching needs 2 time units in our model), cohort 3 of Nymphs 1 and 2; cohort 4 of Nymphs 3 and 4; cohort 5 of Nymphs 5 and cohort 6 of young adult females mating and searching their own territory. We denote by ${c}_{i}(t)$ the size of the *i*-th cohort in time period $t$.

Observe that the developmental time of these cohorts can be considered 5 days approximately (see Table S2 of SI). We also emphasize that the rate of cannibalism does not depend on the size of the offspring, e.g. Nymph 1 and Nymph 2 are attacked with the same probability (${P}_{A}$). When a survived (non-cannibalized) offspring female has matured, it leaves its mother’s territory and is looking for an own territory in the next 5 days. During this migration period it is fertilized. We note that our model focuses on the females assuming that there are males enough for the fertilization of each female. We assume the filial cannibalism rate has effect on neither the migratory behaviour, nor the survival rate during migration. Moreover, we suppose that each new female survives during the migration. Thus from our recursion model the *reproductive season growth rate* of a lineage can be calculated (estimated) if the phenotype in each lineage is fixed.

In our model the numerical response gives the number of hatched new nymphs during our time unite. We call the attention to the fact that the number of laid eggs is not equal to the hatched new nymphs in general, since not all eggs can hatch. Moreover, *N. pseudoferus* is active during the light period of the day, thus we assume 16 h of activity per day. This means that each female is looking for prey 80 h during 5 days. The numerical response is determined by the consumed prey, thus the prey preference (in other words the rate of cannibalism) of the focal female has essential effect on the consumed biomass, and hence on the numerical response too. For the formulation of the numerical response, we start out from the standard modelling methodology of optimal foraging theory, considering the well-known prey preference-dependent Holling type II functional response. Since in our experiment the heterospecific prey density will be fixed, in our model we must assume that the heterospecific prey density (denoted by *y*) is also fixed.

The numerical response depends on the searching time *τ*_*s*_ = 5.3 and the handling time of conspecific and heterospecific prey *τ*_*a*_ = 23.3 and *τ*_*b*_ = 16.6 min, respectively^8^. The probability of a successful attack on different nymph cohorts *κ*_*3*_ = 1.00, *κ*_*4*_ = 0.80, *κ*_*5*_ = 0.55^10^ and the prey preference (denoting by ${P}_{A}$ the conspecific, and by ${P}_{B}$ the heterospecific preference). In our model the prey preference is the probability of an attack when an adult female meets its prey. From the Holling type II functional response we derivate the numerical response by taking into account how many new nymphs can hatch, produced by consuming one nymph belonging to different cohorts (*a*_*3*_ = 0.91, *a*_*4*_ = 3.00, *a*_*5*_ = 6.14, and from one heterospecific prey (*b* = 0.44). We emphasize that we define the phenotypes by the conspecific and heterospecific prey preferences, and we assume that the optimal prey preferences are fixed by evolution. Using numerical methods, in each considered experiment we can calculate the optimal prey preference that maximizes the number of descendants of Eve during one full reproductive season and the life reproductive success during the life span of a focal female.

In Table 1 we visualize how many descendants a focal female of a given phenotype (cannibalism rate) has in her territory. The time unit is 5 days. The first row indicates the “reproduction age” of the focal female, e(*τ*) denotes the number of eggs laid by the focal female at her reproduction age *τ*. In each column the development of the corresponding cohort is given, c_*i*_(*τ*) is the number of descendants in *i*-th stage, that developed from eggs laid by the mother at her reproduction age *τ*. *a*(*τ*) denotes how many adult descendants developed from eggs laid by the mother at her reproduction age *τ*, leave the territory of the mother.

**Table 1:** Biological scheme of our model.

Time	1 (day 5)	2 (day10)	3 (day 15)	4 (day 20)	5 (day 25)	6 (day 30)	7 (day 35)
1 (day 5)	e(1)						
2 (day 10)	e(1)	e(2)					
3 (day 15)	**c**_**1**_**(1)**	e(2)	e(3)				
4 (day 20)	**c**_**2**_**(1)**	**c**_**1**_**(2)**	e(3)	e(4)			
5 (day 25)	**c**_**3**_**(1)**	**c**_**2**_**(2)**	**c**_**1**_**(3)**	e(4)	e(5)		
6 (day 30)	a(1)	**c**_**3**_**(2)**	**c**_**2**_**(3)**	**c**_**1**_**(4)**	e(5)	e(6)	
7 (day 35)		a(2)	**c**_**3**_**(3)**	**c**_**2**_**(4)**	**c**_**1**_**(5)**	e(6)	e(7)
8 (day 40)			a(3)	*c*_*3*_*(4)*	*c*_*2*_*(5)*	*c*_*1*_*(6)*	*e(7)*
9 (day 45)				a (4)	*c*_*3*_*(5)*	*c*_*2*_*(6)*	*c*_*1*_*(7)*
10 (day 50)					*a(5)*	*c*_*3*_*(6)*	*c*_*2*_*(7)*
11 (day 55)						a(6)	*c*_*3*_*(7)*
12 (day 60)							a(7)

Significant values are in bold and italics.

Let us observe that an individual can be cannibalized only after 10 days from birth, since an egg needs 10 days to hatch (see the part highlighted in bold). Cannibalism reduces the number hatched nymphs, but also increases the number of laid eggs. Hence it can be supposed that the focal female lays the least number of eggs during the first ten days of her reproduction period.

For every cannibalism rate, at first step it can be calculated how many nymphs survive cannibalism. (This determines the values c_*i*_(*τ*).) Furthermore, from the number of cannibalised nymphs and the consumed heterospecific prey, using the measured data, based on Holling type II functional response the numerical response can be calculated. The detailed explicit mathematical description of this model calculation can be found in SI B. Finally, there is no cannibalism on nymphs highlighted in italics, since their mothers already died, and we assume the adult individuals leave the territory of their mothers.

Based on Table 1 the descendants tree can be plotted, by means of which it can be calculated how many descendants Eve will have in one reproduction season. (For mathematical details see SI B).

## Results

### Experimental results

The results found in the trial have shown that cannibalism improves the biological parameters of the predatory species. Thus, the development time (Table S2, SI Section a.1.1.2) was significantly shorter for cannibalistic specimens (21.77 ± 0.51 days) than for specimens fed with heterospecific prey (24.11 ± 0.50 days). In turn, the biological parameters of adult females were also influenced by the type of prey (Table S1, SI Section a.1.1.1.2). Adult females fed with conspecifics had a significantly longer oviposition time (33.64 ± 4.00 days) than those fed with heterospecific prey (21.52 ± 2.50 days). Likewise, the fertility of cannibal adult females (89.95 ± 9.84 nymphs/adult female) was significantly higher than in the case of females fed with heterospecific prey (43.74 ± 4.68 nymphs/adult female). This translates into a significantly higher numerical response (0.73 ± 0.09 new nymphs/consumed prey) (0.44 ± 0.05 new nymphs/number of consumed prey) for feeding adult females on conspecific or heterospecific prey, respectively.

### Theoretically, our model can predict filial cannibalism

**Figure 1:**
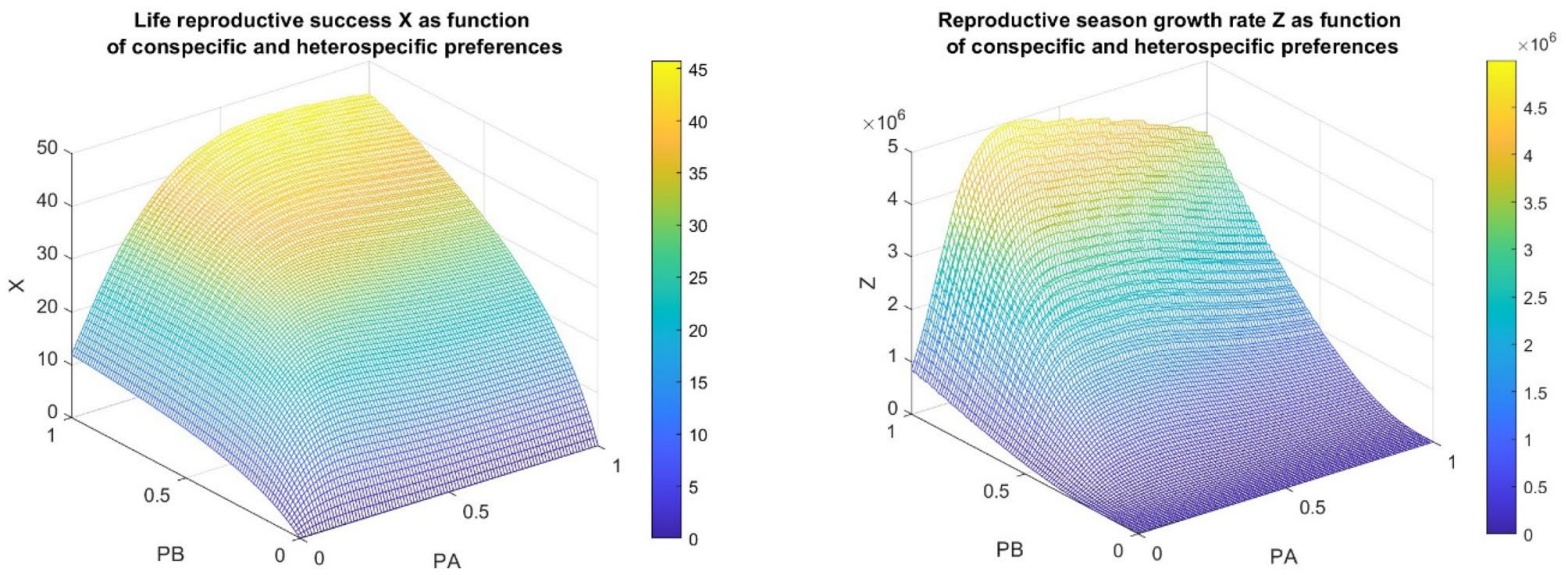
Our model is run with the following illustrative parameters: $y=23.5$*;* $b=0.04$; ${\tau }_{a}=5.0$; ${\tau }_{b}=80.0$; ${\tau }_{s}=30.0$; ${k}_{3}=1$; ${k}_{4}=0.8$; ${k}_{5}=0.55$; ${a}_{3}=2$; ${a}_{4}=6$; and ${a}_{5}=11$. Observe that in this illustrative example of Fig. 1, the conspecific is more valuable food than the heterospecific prey. We numerically found which foraging strategy maximizes the here considered fitness notions. In the left panel, we found that the life reproductive success takes its maximum X(0.6667, 1) = 45.7286, while in the right panel, we found that the reproductive season growth rate (i.e. the number of descendants during one reproductive season) of Eve takes its maximum at different foraging strategies Z(0.3232, 0.9798) = 4.9949E + 6.

First of all, we want to illustrate that our model is appropriate to predict filial cannibalism for both fitness definitions (see Fig. 1). The model simulations are programmed in MATLAB R2023a environment.

### Predictions based on different fitness definitions for the estimated laboratory parameters of *N. pseudoferus*

**Figure 2:**
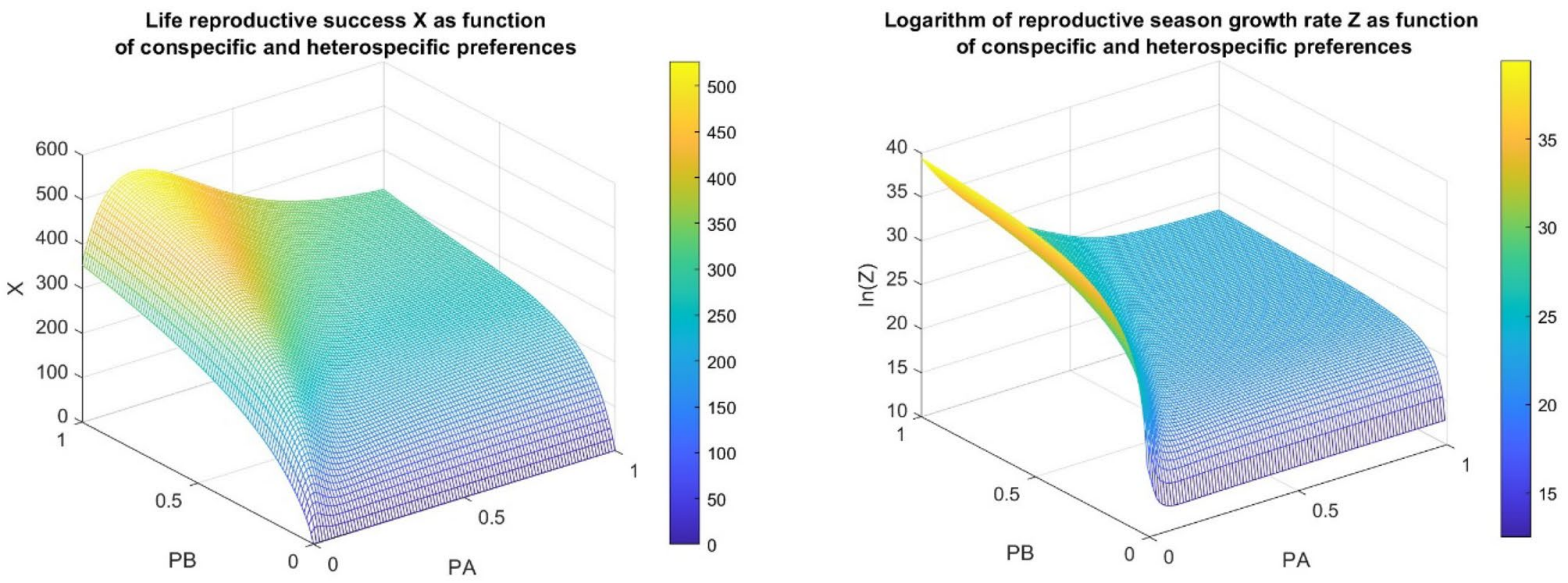
Our model was run with the following real parameters: $y=16.8$; $b=0.44$; ${\tau }_{a}=23.3$; ${\tau }_{b}=16.6$; ${\tau }_{s}=5.3$; ${k}_{3}=1.00$; ${k}_{4}=0.80$; ${k}_{5}=0.55$; ${a}_{3}=0.91$; ${a}_{4}=3.00$; and ${a}_{5}=6.14$: In the left panel of Fig. 2, we calculate the life reproductive success ${\text{X}}$ as a function of different prey preferences. We find that it takes its maximum at the optimal cannibalistic rate ${P}_{A}= 0.1717$ and the optimal heterospecific prey consumption rate ${P}_{B}=1$, with $X\left(0.1717, 1\right)=5.2679$ E $+02.$ In the right panel, we calculate the reproductive season growth rate Z as a function of different prey preferences. We find that the optimal cannibalistic rate is ${P}_{A}= 0$ and the optimal heterospecific prey consumption rate is ${P}_{B}=1$, and the maximum is $Z\left(0, 1\right)= 1.3209$ E $+17.$

Based on the real, laboratory parameters we calculated the optimal foraging strategies for both fitness definitions (see Fig. 2). We found that filial cannibalism of *N. pseudoferus* is an optimal foraging strategy for life reproductive success, but it is not an evolutionarily optimal foraging strategy, since it cannot maximize the descendant’s number at the end of the reproductive season.

## Discussion

For the fitting of the model we set up, we had to measure the parameters of the model. In this work we planned and performed trials to measure only the parameters not measured before, namely, we measured the parameters of the numerical response (see results in “Experimental results” section).

If we want to understand the cannibal behavior of a territorial species from evolutionary aspect, at first step we have to investigate whether cannibalism is optimal from the viewpoint of foraging. Our model is an optimal foraging model based on evolutionary approach, in which to a territorial adult female, along with a heterospecific prey, as a conspecific prey exclusively her own offspring are offered. In our study, as function of food choice, we optimized two fitness concepts: lifetime reproductive success and *reproductive season growth rate* (i.e. the number of descendants of a focal female during one reproductive season). Below we discuss the predictions provided by our model.

Firstly, we demonstrated that our model is appropriate to predict if filial cannibalism is an evolutionarily optimal feeding strategy. Using our mathematical simulation model, we calculated the optimal cannibalism rate of an adult female for two different objective functions. We found that for both fitness definitions, filial cannibalism is an optimal feeding strategy (see Fig. 1), but the optimal values are different according to different fitness definitions. Our theoretical example is based on the collector hypothesis: The offspring accumulate in their bodies a biomass to be consumed, then by their cannibal mother, which using this biomass collected by its own offspring, produces new offspring. This allows the mother to economize much time, since in this way the biomass necessary for laying eggs will be accumulated more quickly. In this way a cannibal mother can produce more eggs in unit time, and as a consequence can increase her numerical response, in comparison to non-cannibal mothers^24^.

Secondly, we have also shown, that the rate of cannibalism of a female maximizing its life reproductive success is higher than that of a female maximizing its descendants number at the end of the reproductive season (see Figs. 1 and 2). The reason for this is that in the maximization of life reproductive success, the developmental time has a role only in a short time period, after that the female has found its territory. We note that now we have a real example for our theoretical result^24,25^.

Thirdly, using our model with the measured laboratory parameters, we calculated the optimal cannibalism rate of an adult female for two different fitness definitions. For the life reproductive success, we found that a partial cannibalism is the optimal feeding strategy. On the contrary, non-cannibalism maximizes the number of descendants at the end of the reproductive season. This means that at the end of the reproductive season the life reproductive success maximizer will have less number of descendants. If we can assume that the survival rates of the cannibal and the non-cannibal individuals are the same during winter, in the long run the life reproductive success maximizer will be eliminated by the number of its descendant maximizer, as we theoretically found earlier^24,25^. We note, that if the cannibal phenotype has radically higher survival rate in winter, then it can win the struggle for life, but this possibility is out of our focus since it is not an optimal foraging reasoning. Furthermore, we have no information about the survival rate during winter.

Based on the above, we can claim that filial cannibalism is not an optimal foraging strategy for *N. pseudoferus* (see Fig. 2) in evolutionary sense. Comparing the real situation with our theoretical example, the main difference between them is that the numerical response coefficient for a *N. pseudoferus* nymph is too small. Namely, a cannibal mother can consume its own offspring from cohort 1 with high success (${k}_{3}=1$), on average she will have less than one $({a}_{3}=0.91$) newborn nymph from it.

There are the following two evolutionary costs of filial cannibalism:

*Time delay effect*: The new eggs (produced from the consumed sibling biomass) need time for the complete development, while the consumed nymphs could have started reproducing earlier. In other words, the cannibalism can increase the egg number, but the cannibalistic lineage wastes time, since the individuals hatched from eggs produced by cannibalism start to reproduce later.

*Lineage elimination effect:* When a female consumes an own nymph, not only one female offspring is eliminated, but also all potential descendants from the cannibalized female offspring during the rest of reproductive season.

In the cases where cannibalism is an evolutionarily optimal foraging strategy, the above two effects certainly should be taken into account, i.e., the cannibal mother not only should produce more than one newborn offspring, but these offspring should also compensate the reduction of the long-term rate of increase, caused by both effects. We note that, the numerical response coefficient obtained in our laboratory trials was so small, that there is no hope to get a positive result by refining our deterministic model.

Our result once again calls attention to the fact that, in case of overlapping generations, the concept of adaptivity can be explained only involving the following two issues: 1. At individual level (in terms of life reproductive success), which behavior can be considered more favourable^28^. 2. Which behavior maximizes the number of descendants? By means of theoretical investigation we have already shown that the answers to both questions are not necessarily the same^24,25^. In the present work we gave a real example to support our theoretical result.

In the present paper we had to decide the hypothesis that the maternal cannibalism is purely optimal foraging strategy. Now that the answer is negative, in the future we will be aimed at looking for further possible reasons. We conjecture that the survival of the decrease in the local density of the heterospecific prey can be substantially modified by the familial cannibalism. This may be one of the possible reasons for the maternal cannibalism to be adaptive in the case of a territorial female. We would like to study this in a following paper.

Finally, it is well known that *N. pseudoferus* is a cannibal species^10^. Now we can exclude the first theoretical possibility for filial cannibalism of *N. pseudoferus*, since its filial cannibalism is not an evolutionarily optimal foraging strategy. Based on this result, we can already focus our study on other advantages of cannibalism known in the literature, such as *e.g.*, longer longevity or survival during critical time^4,29,30^.

### Supplementary Information

Supplementary Information.

## Data Availability

The data that support the main findings of this study are publicly available at http://hdl.handle.net/10835/16272.
