# Supplementary material for: Filial cannibalism of Nabis pseudoferus is not evolutionarily optimal foraging strategy
Source: Sci Rep. 2024 Apr 19;14:9022. doi: 10.1038/s41598-024-59574-7 (PMC11031581; doi:10.1038/s41598-024-59574-7)
Supplement: Supplementary file 1 — Supplementary Information. [file 41598_2024_59574_MOESM1_ESM.docx]

**SUPPLEMENTARY INFORMATION**

**Filial cannibalism of *Nabis pseudoferus* is not evolutionarily optimal foraging strategy**

József Garay, Manuel Gámez, Yohan Solano-Rojas, Inmaculada López, Ana Belén Castaño-Fernández, Zoltán Varga, Tamás F. Móri, Villő Csiszár, Tomás Cabello

**A) Materials and methods of experiment**

**a.1) EXPERIMENTAL PART**

The data used in the recursion were obtained, on the one hand, from the results of the test carried out as described in Section a.1.1.1 below and the parameters are calculated in Section a.1.1.2. On the other hand, the other parameters also used in the recursion are shown in Section a.1.1.3.

**a.1.1. Effects of cannibalism on predator biology**

a.1.1.1- Materials and methods

- *Biological materials*:

All insects used in the trial came from populations maintained in the Applied Entomology Laboratory, Department of Biology and Geology, University of Almeria. They were maintained under controlled conditions at 25 ± 1ºC. 65 ± 10% RH and 16:8 h photoperiod (L:O). The predatory *N. pseudoferus* was reared following the methodology described by^1,2^, for more than 30 generations, and fed with eggs (as factitious prey) of the Mediterranean flour moth. *Ephestia kuehniella* Zeller (*Lepidoptera: Pyralidae*) and green bean pods (*Phaseolus vulgaris* L.), as a water source for nymphs and adults and as oviposition substrate. These eggs were supplied frozen (Ephescontrol®. Agrobio S.L., La Mojonera, Almeria. Spain) and kept at - 40 °C until use.

Periodically, specimens of the II- and III-instar nymphs of *N. pseudoferus* were collected from these laboratory populations and preserved under freezing at -40°C until their use in the assay as conspecific prey. In turn, the laboratory population of *S. exigua* (Hübner) (*Lepidoptera: Noctuidae*) was established from a population of larvae collected from a commercial pepper (*Capsicum annuum* L.) crop in commercial greenhouses (36.721796º N, 2.730339º S) in Almeria, Spain. The larvae were transferred to the laboratory to be reared individually in 25 ml capacity containers on artificial diet following the methodology described by^3,4^; subsequently, the diet composition was slightly modified by^5^. Also, periodically, specimens of the II- and III-instar larvae of *S. exigua* were collected and preserved under freezing at -40 ºC until their use in the trial as heterospecific prey.

Prior to the trial, the fresh weights of the larvae (I- and II-instar) of the heterospecific prey (*S. exigua*) and those of the nymphs (I- to V-instar) for the conspecific species (*N. pseudoferus*) were evaluated. For this purpose, at least 100 eggs, chosen at random, were taken from the laboratory population of *S. exigua* which were isolated in containers (25 ml) until the emergence of larvae that were subsequently fed as above with artificial diet. For the predator species, bean pieces were selected in which eggs had been inserted and these were isolated in a container (100 ml) until about 100 specimens emerged; later, when the neonate nymphs emerged, these were isolated and fed as indicated above with eggs of *E. kuehniella*. All specimens of both species were evolved in the laboratory under the above conditions. Also, in each species, the molted specimens were observed and weighed within 24 hours of molting. A precision balance was used for this purpose (model AUW120D semi-micro analytical balance, 42g/0.01mg; Shimadzu Corporation, Kyoto, Japan).

-*Experimental Design and Procedures*:

The experimental design was completely randomized with one factor (prey species at two levels: conspecific and heterospecific prey) with a variable number of replications. The dependent variable was the different values of the life cycle of the adult female predator and its offspring until the next generation, as described below.

The life cycle of the predator *N. pseudoferus* was studied under controlled conditions (25 ± 1 ºC. 65±10% RH and 16:8 h L:O) in ICP-600 incubators (Memmert®. Schwabach. Germany). Newly formed females and males (0 - 24 h of age) were collected from the laboratory populations and placed, in pairs, in plastic containers (100 cm^3^) and closed with a perforated lid to allow ventilation. The males were left together with the females for a period of 24 h. with the purpose of allowing mating, but minimizing possible cannibalism. For this purpose, during this first week, the amount of food (factitious prey: *E. kuehniella*) was doubled (2 x 16 mg in one week) to provide enough food for females and males. After this period, the males were removed, and the females continued to be individualized and fed with the respective prey for each treatment.

The treatments consisted of providing 16.0 mg of food/week, which corresponds to enough food for the maintenance of predator adults per week (unpublished data). This amount was dosed according to each prey as follows, previously defrosted: the II- and III-instar of *N. pseudoferus* were offered in the amount of 8.0 mg twice per week; in turn. the II- and III-instar of *S. exigua* were offered in the amount of 4.0 mg four times per week. These differences were because the prey offered retained their organoleptic characteristics and suitability as fresh food to be consumed by adult females (unpublished data). After replacing the food offered to each female, a count of the prey consumed or not, was carried out under binocular microscopy.

Likewise, each female was offered a piece of bean pod, twice a week, as a source of water and oviposition substrate. These bean pods, after being replaced, were isolated in a new plastic container identical to the one described above, until the emergence of the neonate nymphs. Weekly, 25 eggs from different females and treatments were randomly selected and individually isolated in a plastic container (100 cm^3^). After hatching, the neonate nymphs were fed the same type, quantity, and frequency of food (prey species) offered to the mothers. These immature specimens were evolved until the emergence of the new adults; they were evaluated twice a week to determine the time of each molt. as well as the prey consumed. Finally, when they reached the adult stage, they were sexed, previously fixed, by means of the structure of the external genitalia.

-*Recorded data and statistical analysis*:

For the immature stages, the duration of the egg stage was recorded, as indicated above, as well as the duration of the 5 nymphal stages, in each treatment. For the adult female, the duration of the pre-ovipositional, ovipositional, and post-ovipositional periods were recorded; as well as the number of eggs oviposited on the substrate per adult female, in each treatment, and the number of viable eggs upon emergence of neonate nymphs from these eggs, as indicated above. These data were analyzed using Generalized Linear Models (GLMZ) using IBM SPSS statistical^6^. In each analysis the significance of the model was assessed by an Omnibus test, which allowed to evaluate whether the variance of the explained data set was significantly greater than the unexplained variance. The Wald statistical test was used to pairwise compare the mean values of the treatments.

a.1.1.2.- Experimental results: Parameters used in recursion

-*Reproductive period for adult females*: The total longevity, as well as its division into periods, is shown in the same for females of *N. pseudoferus* are shown in Table S1.

| **Value** | **Prey** | **Average** | **S.E.** | **Statistical parameters** | | | |
| --- | --- | --- | --- | --- | --- | --- | --- |
|  |  |  |  | **N** | ***χ^2^*** | **d.f.** | ***P*** |
| Preoviposition period (days) | *S. exigua* | 7.17a | ±0.30 | 23 | 6.470 | 1 | *P* < 0.05 |
|  | *N. pseudoferus* | 6.14a | ±0.30 | 22 |  |  |  |
| Oviposition period  (no. alive nymphs per female) | *S. exigua* | 21.52 | ±2.50 | 23 | 6.749 | 1 | *P* < 0.01 |
|  | *N. pseudoferus* | 33.64 | ±4.00 | 22 |  |  |  |
| Post ovipositional period (days) | *S. exigua* | 5.46 | ±0.76 | 13 | 0.117 | 1 | n.s. |
|  | *N. pseudoferus* | 5.09 | ±0.77 | 11 |  |  |  |
| Total longevity (days) | *S. exigua* | 31.74 | ±2.48 | 23 | 5.483 | 1 | *P* < 0.05 |
|  | *N. pseudoferus* | 41.55 | ±3.32 | 22 |  |  |  |
| (*) Values for value pairs with different letter show significant differences at *P* = 0.01. | | | | | | | |

**Table S1**. Reproductive period for adult females of Nabis pseudoferus when fed with conspecific or heterospecific prey under laboratory conditions (at 25±1 ºC. 80-60% R.H. and 16:8 hours Day:Night) (*).

The reproductive period of adult females was 33.64±3.98 days feeding on conspecific prey and significantly longer than on heterospecific prey (21.50±2.49 days). This value used in the recursion is within the actual limits obtained in the experimental test.

-*Duration of immature stages*: Values for the durations of the different predator immature stages used are shown in Table S2.

| **Stage** | **Prey** | **Average** | **S.E.** | **Statistical parameters** | | | |
| --- | --- | --- | --- | --- | --- | --- | --- |
|  |  | **(days)** | | **N** | ***χ^2^*** | **d.f.** | ***P*** |
| Egg | - | 8.44a | ±0.10 | 25 | - | - | n.s. |
|  | - | 8.44a | ±0.10 | 25 |  |  |  |
| I-instar | *S. exigua* | 2.70a | ±0.14 | 25 | 0.696 | 1 | n.s. |
|  | *N. pseudoferus* | 2.50a | ±0.14 | 25 |  |  |  |
| II-instar | *S. exigua* | 2.01a | ±0.11 | 24 | 2.020 | 1 | n.s. |
|  | *N. pseudoferus* | 2.23a | ±0.11 | 25 |  |  |  |
| III-instar | *S. exigua* | 2.20a | ±0.08 | 22 | 10.103 | 1 | < 0.01 |
|  | *N. pseudoferus* | 1.81b | ±0.09 | 25 |  |  |  |
| IV-instar | *S. exigua* | 2.64a | ±0.13 | 22 | 0.123 | 1 | n.s. |
|  | *N. pseudoferus* | 2.70b | ±0.13 | 24 |  |  |  |
| V-instar | *S. exigua* | 6.16a | ±0.39 | 22 | 10.811 | 1 | < 0.01 |
|  | *N. pseudoferus* | 4.27b | ±0.38 | 21 |  |  |  |
| Total nymph | *S. exigua* | 15.71a | ±0.47 | 22 | 11.063 | 1 | < 0.01 |
|  | *N. pseudoferus* | 13.34b | ±0.48 | 21 |  |  |  |
| Total development | *S. exigua* | 24.11a | ±0.50 | 22 | 9.602 | 1 | < 0.01 |
|  | *N. pseudoferus* | 21.77b | ±0.51 | 21 |  |  |  |
| (*) Values for value pairs with different letter show significant differences at *P* = 0.01. | | | | | | | |

**Table S2**. Average (±SE) of the developmental time of immature stages of Nabis pseudoferus when fed with conspecific or heterospecific prey under laboratory conditions (at 25±1 ºC. 80-60% R.H. and 16:8 hours Day:Night) (*).

According to Table S2, the following time periods were considered in the model (for cohorts): eggs (cohort 1 and 2); nymphal instars N-I and N-II (cohort 3); nymphal instars N-III and N-IV (cohort 4); nymphal instar N-V (cohort 5); and young adult females (cohort 6). Each with a duration of 5 days; except egg-01 which was considered 0 (due to overlaps in the development of the species with overlapping generations)

-*Numerical response*:

The fresh prey of the different stages of both insect species used in the assay were as follows: For the larvae of the heterospecific species S. exigua they were 0.00039±0.00001 g, and 0.00601±0.00021 g for the II- and III-instar, respectively. In turn, for the conspecific species the fresh weights of the nymphal stages were 0.000441±0.000082 g, 0.000802±0.000182 g, 0.001474±0.000362 g, 0.002607±0.000655 g, 0.004178±0.000436 for I-, II-, III-, IV-, and V-instar, respectively.

Table S3 shows the numerical response of predator females fed with conspecific and heterospecific prey expressed as number of nymphs hatched according to the weight (mg) of prey consumed.

| **Value** | **Prey** | **Average** | **S.E.** | **Statistical parameters** | | | |
| --- | --- | --- | --- | --- | --- | --- | --- |
|  |  |  |  | **N** | ***χ^2^*** | **d.f.** | ***P*** |
| Total consumed prey  (no.) | *S. exigua* | 106.55a | ±8.31 | 23 | 5.483 | 1 | P < 0.05 |
|  | *N. pseudoferus* | 139.47b | ±11.13 | 22 |  |  |  |
| Total fertility  (no. alive nymphs per female) | *S. exigua* | 43.74a | ±4.68 | 23 | 18.111 | 1 | P < 0.01 |
|  | *N. pseudoferus* | 89.95b | ±9.84 | 22 |  |  |  |
| Numerical response (nymphs/prey) | *S. exigua* | 0,44a | ±0.05 | 23 | 8.138 | 1 | P < 0.01 |
|  | *N. pseudoferus* | 0,74b | ±0.09 | 22 |  |  |  |
| (*) Values for value pairs with different letter show significant differences at *P* = 0.01. | | | | | | | |

**Table S3.** Average (±SE) of the total consumed prey, total fertility (no. of live nymphs), and numerical response of adult female Nabis pseudoferus when fed with conspecific or heterospecific prey under laboratory conditions (at 25±1 ºC. 80-60% R.H. and 16:8 hours Day:Night) (*).

From the above data we calculated the following parameters:

1. Parameter '*Y*' (average number of heterospecific prey consumed per week): according to the data obtained in the trial the average total number of heterospecific prey consumed per predator female was 106.55 heterospecific prey (Table S3) for an oviposition period of these of 31.5 days (= 4.50 weeks) (Table S1), then, *Y* = 16.8 heterospecific prey consumed per week.
2. Parameter '*b*' (represents numerical response of adult females of *N. pseudoferus* fed with the heterospecific prey *S. exigua* taking into account the nymphs hatched per prey consumed): This value showed a value of *b* = 0.44±0.05 (Table S3).
3. Parameters '*a_i_*' represent estimates of the numerical response of adult *N. pseudoferus* females fed on conspecific prey when they are at different age stages:

The numerical response (N.R.) for conspecific prey = 0,73±0.09 (Table S3), The 1/NR is the number of alive nymphs produces per 1 unity of conspecific prey = 1.36 new alive nymphs. For this prey (larval instar N-II and N-III), we estimated that the mean unit weight of the prey offered and consumed is to 0.000926 g.

Then, we estimate the *a_i_* values as follows:

-For cohort 3 (Age class: N-1 and N-2):

As the average biomass per unit of prey of the class is ω_3_ = (0,000441+0,000802)/2 = 0,000622 g, we estimated the number of nymphs produced for this biomass (ω_3_) as *a_3_* = 1469.97 *0,000622 = 0.91 No. of nymphs/No. of prey.

-For cohort 4 (Age class N-3 and N-4):

-ω_4_ (average biomass per unit of prey) = (0,001474+0,002607)/2 = 0,0020405 g,

Then *a_4_* = 1469.97*0.0020405 = 3.00.

-For cohort 5 (Age class N-5):

The average biomass of this class is (ω_5_) = 0.0041781 g,

Then, *a_5_* = 1469.97*0.0041781 = 6.14.

a.1.1.3.- Other parameters used in the recursion

(iv) Parameter *τ_a_*, *τ_b_*, and *τ_s_*:

These values were obtained from the publication of^1^ in their Table S2 representing the values of handling times (*T_h_*) for conspecific prey (*τ_a_* = 23.3±3.3 minutes) and for heterospecific prey (*τ_b_* = 16.6±2.5 minutes) in a non-choice test. In turn, the value of the search time (*T_s_*) for heterospecific prey (*τ_s_* = 5.3±1.9 minutes in the same type of non-choice test.

(v) Parameters *k_i_*

The *k_i_* parameters were obtained from data published, in turn, by^2^ converted to the survival rate of *N. pseudoferus* nymphs when exposed, for 72 hours, to an adult female of the same species (Table S4).

| **Predator**  **stage** | **Prey stage** | | | | | |
| --- | --- | --- | --- | --- | --- | --- |
|  | **Survivors (proportion) in 72 hours** | | | | | |
|  | Adult | V-instar | IV-instar | III-instar | II-instar | I-instar |
|  |  |  |  |  |  |  |
| Adult | 0.65 | 0.45 | 0.25 | 0.20 | 0.00 | 0.00 |

**Table S4**. Survival values of the different stages of Nabis pseudoferus subjected to cannibalism, for a maximum period of 72 hours, recalculated from Fernandez et al (2020).

In the recursion *κ_3_* = 1, *κ_3_* is the killing rate of the first age class of nymphs (*i.e.*, I- and II-instars). *κ_4_* ≈ 1-0.2 = 0.8, *κ_4_* is the killing rate of the second age class of nymph (*i.e.*, III- and IV-instars). *κ_5_* ≈ 1-0.45=0.55, *κ_5_* is the killing rate of the third age class of nymphs (*i.e.* V-instar).

**References**:

1. Fernandez, F.J. *et al.* Cannibalism: Do risks of fighting and reprisal reduce predatory rates? *Community Ecol.* **18**(1), 87–96; [10.1556/168.2017.18.1.10](https://doi.org/10.1556/168.2017.18.1.10) (2017).
2. Fernandez, F.J., Gámez, M., Garay, J. & Cabello, T. Do development and diet determine the degree of cannibalism in insects? To eat or not to eat conspecifics. *Insects*. **11**,242; [10.3390/insects11040242](https://doi.org/10.3390/insects11040242) (2020).
3. Cabello, T., Rodríguez, H. & Vargas, P. Development. longevity and fecundity of *Spodoptera littoralis* (Lep.: Noctuidae) reared on eight artificial diets. *J. Appl. Entomol.* **97**, 494–499; [10.1111/j.1439-0418.1984.tb03781.x](https://doi.org/10.1111/j.1439-0418.1984.tb03781.x) (1984).
4. Cabello, T., Rodriguez, H. & Vargas, P. Utilización de una dieta artificial simple en la cría de *Heliothis armigera*. *Spodoptera littoralis* y *Trigonophora meticulosa* (Lep. Noctuidae). *Anales del Instituto Nacional de Investigaciones Agrarias*. Serie: Agrícola. **27**, 101–107 (1984).
5. Amate, J., Barranco, P. & Cabello, T. Biología en condiciones controladas de especies de noctuidos plaga (Lep.: Noctuidae). *Bol. San. Veg. Plagas*. **26**, 193–201(2000).
6. IBM Corp. Released *IBM SPSS Statistics for Windows*. Version 28.0. Armonk. NY: IBM Corp. (2021).

**B) Theoretical model**

Our aim is to build up a mathematical model, which is as near as possible to the biological characterization of *Nabis* and our experiments. To do this, we have to take account of two main points: the prey preference dependent numerical response, and the life history of *Nabis*.

**Notation**

Life history of a female:

egg → nymph I → nymph II → nymph III → nymph IV → nymph V → young adult ­→ reproductive adult,

with the following durations

egg: 10 days

nymph I: 2.1 days

nymph II: 1.7 days

nymph III: 2.9 days

nymph IV: 2.1 days

nymph V: 4.5 days

young adult: 5 days

reproductive adult: further 35 days.

The time period of population growth is set to $T=5$ days. This is considered as the time unit. In order to deal with integer multiples of the time unit we consider the following cohorts (each corresponding to one time period):

The egg state is divided into two: cohort 1 and cohort 2. States nymph I and nymph II form cohort 3, nymph III and nymph IV form cohort 4; while states nymph 5 and young adult form distinct cohorts (cohorts 5 and 6).

$c_{1}\left( t \right), t=1, 2,\ldots$ – number of eggs laid in time period $t$ (cohort 1)

$c_{2}\left( t \right), t=1, 2,\ldots$ – number of eggs of cohort 2 in time period $t$

$c_{3}\left( t \right), t=1, 2,\ldots$ – size of cohort 3 at the beginning of time period $t$ (before filial cannibalism)

$c_{4}\left( t \right), t=1, 2,\ldots$ – size of cohort 4 at the beginning of time period $t$

$c_{5}\left( t \right), t=1, 2,\ldots$ – size of cohort 5 at the beginning of time period $t$

$c_{6}\left( t \right), t=1, 2,\ldots$ – size of cohort 6 at the beginning of time period $t$ (young adult females mating and searching their own territory)

$x\left( t \right), t=1, 2,\ldots$ – adult female offspring in time period $t$

$P_{A}$ – recognition probability preference of conspecific nymph (prey A)

$P_{B}$– recognition probability preference of heterospecific prey

*κ_3_*, *κ_4_*, *κ_5_* – probability that a nymph in the corresponding cohort gets killed by the adult when met

*a_3_*, *a_4_*, *a_5_* – biomass of unit prey A in the corresponding cohort (how many eggs it contributes to)

$b$ – biomass of unit prey B

*τ_a_*, *τ_b_*, *τ_s_* – average time of searching, handling prey A, and handling prey B, respectively (in minutes)

$y$ – density of prey B (average number of prey B in the perception range). Considered constant.

*δ_t_*, *t* = 1, 2,…– indicator of mother’s being alive: *δ_t_* = 1 for 1 ≤ t ≤ 7, and 0 otherwise.

What happens during a five-day period?

Suppose that a certain moment there are $w_{3}$, $w_{4},$ $w_{5}$ nymphs of age classes $3$, $4$ and $5$, resp. Then, after a searching time of length $\tau_{s}$, the following mutually exclusive random events are possible.

- *Nabis* finds and recognizes a heterospecific prey, with probability

$$\frac{P_{B}y}{w_{3}+w_{4}+w_{5}+y} .$$

Then the biomass she gains is $b$, and an additional handling time $\tau_{b}$ is needed before the next searching period. Quantities $w_{3}$, $w_{4}$, $w_{5}$ and $y$ remain unchanged.

- *Nabis* finds, recognizes, and kills a conspecific nymph of age class 3, with probability

$$\frac{P_{A}\kappa_{3}w_{3}}{w_{3}+w_{4}+w_{5}+y} .$$

Then the biomass she gains is $a_{3}$, and an additional handling time $\tau_{a}$ is needed before the next searching period. Quantities $w_{4}$, $w_{5}$ and $y$ remain unchanged, $w_{3}$ decreases by $1$.

- *Nabis* finds, recognizes, and kills a conspecific nymph of age class 4, with probability

$$\frac{P_{A}\kappa_{4}w_{4}}{w_{3}+w_{4}+w_{5}+y} .$$

Then the biomass she gains is $a_{4}$, and an additional handling time $\tau_{a}$ is needed before the next searching period. Quantities $w_{3}$, $w_{5}$ and $y$ remain unchanged, $w_{4}$ decreases by $1$.

- *Nabis* finds, recognizes and kills a conspecific nymph of age class 5, with probability

$$\frac{P_{A}\kappa_{5}w_{5}}{w_{3}+w_{4}+w_{5}+y} .$$

Then the biomass she gains is $a_{5}$, and an additional handling time $\tau_{a}$ is needed before the next searching period. Quantities $w_{3}$, $w_{4}$ and $y$ remain unchanged, $w_{5}$ decreases by $1$.

- The searching period is not successful (*Nabis* finds but cannot recognize or kill the prey). There is neither gain, nor additional waiting time. The probability of this event makes up the sum of the probabilities of the first four possibilities to 1.

Thus, after a searching (and possibly handling) period, the expected gain is

$$\frac{P_{A}\left( a_{3}\kappa_{3}w_{3}+a_{4}\kappa_{4}w_{4}+a_{5}\kappa_{5}w_{5} \right)+P_{B}by}{w_{3}+w_{4}+w_{5}+y} ,$$

the expected additional handling time is

$$\frac{\tau_{a}P_{A}\left( w_{3}+w_{4}+w_{5} \right)+\tau_{b}P_{B}y}{w_{3}+w_{4}+w_{5}+y} ,$$

while the expected quantities of conspecific nymphs of age classes $3$, $4$ and $5$ are

$$w_{3}\left( 1-\frac{P_{A}\kappa_{3}}{w_{3}+w_{4}+w_{5}+y} \right),$$

$$w_{4}\left( 1-\frac{P_{A}\kappa_{4}}{w_{3}+w_{4}+w_{5}+y} \right),$$

$$w_{5}\left( 1-\frac{P_{A}\kappa_{5}}{w_{3}+w_{4}+w_{5}+y} \right),$$

respectively ($b$ remains unchanged).

Now the recursion is as follows.

**Variables.** $P_{A}\in\left[ 0, 1 \right], P_{B}\in[0, 1]$ (also called strategies)

**Recurrence relation**

**input:**  $P_{A}, P_{B}$ % recognition probabilities

**output:** $x\left( t \right), 1\leq t\leq50$ % the length of the reproductive season

% is about $250$ days $= 50$ time units

**initial condition**: $c_{2}\left( 1 \right)=c_{3}\left( 1 \right)=c_{4}\left( 1 \right)=c_{5}\left( 1 \right)=c_{6}\left( 1 \right)=x\left( 1 \right)=0.$

**for** $t=1$ **to** 49 **do**

**if** $1\leq t\leq7$ **then**

$w_{3}:=c_{3}\left( t \right), w_{4}:=c_{4}\left( t \right), w_{5}:=c_{5}\left( t \right)$,

$K:=0$ % cumulative time

$G:=0$ % cumulative gain

**while** $K < 4800$ % $4800$ is the length of the active period

% (measured in minutes).

$G:=G+\frac{P_{A}\left( a_{3}\kappa_{3}w_{3}+a_{4}\kappa_{4}w_{4}+a_{5}\kappa_{5}w_{5} \right)+P_{B}by}{w_{3}+w_{4}+w_{5}+y}$

$w_{3}:=w_{3}\left( 1-\frac{P_{A}\kappa_{3}}{w_{3}+w_{4}+w_{5}+y} \right)$

$w_{4}:=w_{4}\left( 1-\frac{P_{A}\kappa_{4}}{w_{3}+w_{4}+w_{5}+y} \right)$

$w_{5}:=w_{5}\left( 1-\frac{P_{A}\kappa_{5}}{w_{3}+w_{4}+w_{5}+y} \right)$

$K:=K+\tau_{s}+\frac{\tau_{a}P_{A}\left( w_{3}+w_{4}+w_{5} \right)+\tau_{b}P_{B}y}{w_{3}+w_{4}+w_{5}+y}$

**end while**

$c_{2}\left( t+1 \right):=G$

$c_{3}\left( t+1 \right):=c_{2}(t)$

$c_{4}\left( t+1 \right):=w_{3}$

$c_{5}\left( t+1 \right):=w_{4}$

$c_{6}\left( t+1 \right):=w_{5}$

$x\left( t+1 \right):=c_{6}(t)$

**else**

$c_{2}\left( t+1 \right):=0$

$c_{3}\left( t+1 \right):=c_{2}(t)$

$c_{4}\left( t+1 \right):=c_{3}(t)$

$c_{5}\left( t+1 \right):=c_{4}(t)$

$c_{6}\left( t+1 \right):=c_{5}(t)$

$x\left( t+1 \right):=c_{6}(t)$

**end if**

**end for**

**return** $x\left( t \right), 1\leq t\leq50$

**Objective functions**

The total number $X$ of (adult female) offspring of the first female called *Eve* (who enters the reproductive adult age at the beginning of the reproductive season) is her *life reproductive success.* Clearly,

$$X=\sum_{t=1}^{13} x(t).$$

On the other hand, *evolutionary success* can be measured by the total number of living descendants of the first female, Eve, at the end of the reproductive season. Let $z(t)$ denote the number of all (adult female) descendants of Eve alive at the end of time period $t$ (possibly including herself, that is, we consider the family size). This quantity satisfies the following recursion.

$$z\left( 1 \right)=1, z\left( t \right)=\delta_{t}+\sum_{i=2}^{t} x\left( i \right) z\left( t+1-i \right), t\geq2.$$

Then, evolutionary success is measured by $Z=z(50)$.

**Aim.** We have to find the maximum of $X$ and $Z$ together with the maximizing strategies $P_{A}$ and $P_{B}$ .

**Parameters**

Theoretical example:

$y=23.5$

$b= 0.04$

$\tau_{a}=5, \tau_{b}=80, \tau_{s}=30$

$\kappa_{3}=1, \kappa_{4}=0.8, \kappa_{5}=0.55$

$a_{3}=2, a_{4}=6, a_{5}=11$

Experimental example (*Spodoptera exigua*-*Nabis pseudoferus*):

$y=16.8$

$b= 0.44$

$\tau_{a}=23.3, \tau_{b}=16.6, \tau_{s}=5.3$

$\kappa_{3}=1, \kappa_{4}=0.8, \kappa_{5}=0.55$

$a_{3}=0.91, a_{4}=3.00, a_{5}=6.14$
